# Spin-Lattice Distribution MRI Maps Nigral Pathology in Progressive Supranuclear Palsy (PSP) during Life: A Pilot Study

**DOI:** 10.1371/journal.pone.0085194

**Published:** 2014-01-28

**Authors:** Michael Hutchinson, Ulrich Raff, Pedro Chaná, Isidro Huete

**Affiliations:** 1 Department of Neurology, NYU Langone School of Medicine, New York, New York, United States of America; 2 Department of Physics and Medicine, University of Santiago de Chile, Santiago, Chile; 3 CETRAM, University of Santiago de Chile, Santiago, Chile; 4 Department of Radiology, Catholic University of Chile School of Medicine, Santiago, Chile; INSERM Paris, France</address>

## Abstract

An MRI biomarker for Parkinsonism has long been sought, but almost all attempts at conventional field strengths have proved unsatisfactory, since patients and controls are not separated. The exception is Spin-Lattice Distribution MRI (SLD-MRI), a technique which detects changes in the substantia nigra (SN) due to changes in the spin-lattice relaxation time, T_1_. This easily separates patients with Parkinson's disease (PD) from control subjects at 1.5 Tesla, suggesting that it may be sensitive to presymptomatic disease. SLD-MRI demonstrates a topography of *signal* change within the SN which is the same as the known topography of *pathological* change, where the lateral portions of the nucleus are more affected than the medial. In a further step towards its validation, we apply SLD-MRI to a disease control, Progressive Supranuclear Palsy (PSP), the most common of the atypical forms of Parkinsonism. In PSP the topography of pathological change in the SN is reversed. We therefore hypothesized that PSP would show a topography of SLD-MRI *signal* change in the SN that is the reverse of PD (i.e. the medial portion is more affected than the lateral). All 7 patients showed such a topography of MR signal, and all patients were separated from control subjects. Although this is a step toward validation of SLD-MRI with respect to sensitivity and disease specificity, nevertheless we stress that this is a pilot project only. Validation will only be possible when comparing larger cohorts of PSP, PD and control subjects.

## Introduction

MRI is known for its exquisite sensitivity to acute and subacute pathology. This is because T_2_-weighted images are sensitive to small changes in the amount and character of water content in tissue. When it comes to chronic neurodegenerative conditions, where tissue content of water may not change, conventional MRI pulse sequences are much less sensitive to micropathology.

In the last 25 years there have been over 100 papers describing attempts to make MRI sensitive to Parkinson's Disease (PD). The extent of this literature suggests both the importance of the endeavor, and also a tacit dissatisfaction with existing nuclear medicine techniques. Yet nuclear techniques, despite their relatively high cost, limited availability, and substantial exposure to ionizing radiation, have remained the gold standard for detection.

Given its enormous utility in other contexts, nevertheless, it is hardly surprising that there should have been a large effort to make MRI sensitive to PD. The techniques previously investigated include the following: spin-spin relaxation (T_2_ and T_2_^*^), Magnetization Transfer Imaging (MTI), Diffusion-sensitive imaging (DTI and FA), spectroscopy, volumetric assays of SN volume, and spin-lattice relaxation (T_1_). Recently there has been an extensive review by Lehericy *et al* [1].

As a rule, MRI at conventional field strengths has not been particularly useful as a biomarker for PD, since patients and control subjects are not separated. The existing techniques therefore lack sensitivity and specificity for PD, and cannot reliably detect presymptomatic disease. This has now led to increasing efforts to investigate MRI at higher field strengths.

There is an exception to this rule, however, a supercharged T_1_-weighted technique [2]–[6], now in an automatic form known as spin-lattice distribution imaging (SLD-MRI) [7]. This technique depends on changes in the spin-lattice relaxation time which occur with neurodegeneration [7]. Presumably “the lattice” is composed mainly of the macromolecular intraneuronal milieu, and when there are gross changes in this milieu, as with neurodegenerative change, there will be changes in T_1_.

The technique employs a unique ratio of two inversion recovery ratio images of the SN, Gray Matter Suppressed (GMS), and White Matter Suppressed (WMS). After the SN is automatically extracted from the background, the ratio image of the SN is denoted R  =  WMS/GMS. R demonstrates a topography of *signal* change in the SN corresponding to the known gradient of *pathological* change, i.e. from lateral-to-medial.

We used the identical technique to image the SN in Progressive Supranuclear Palsy (PSP), a form of Parkinsonism where the topography of pathological change in the SN is known to be opposite to that in PD i.e. the medial portion of the nucleus being more affected than the lateral.

First described in 1963, the most common of the atypical forms of Parkinson's disease is now usually referred to as PSP [8]. It is a progressive, bradykinetic, adult-onset neurodegenerative disorder which superficially resembles PD. However it is clinically distinct from PD in that there is a striking restriction of vertical gaze, and truncal rigidity which is greater than limb rigidity. Tremor is usually less prominent than in PD, and there is no asymmetry. In addition there is usually either no response, or only a minimal response, to levodopa.

Nevertheless, given that both PD and PSP are associated with bradykinesia, rigidity and postural instability, and given that PD often presents without tremor, it is not surprising that PSP is often misdiagnosed as PD [9].

Pathologically, PSP is characterized by gliosis and neuronal cell loss, with tau protein, in the SN, with the most severe changes occurring in the medial portion of the SN [10]. This is opposite to the topography of pathological change seen in PD. Presumably the truncal-greater-than-limb rigidity arises from this unique topography, just as PD demonstrates limb-greater-than-trunk rigidity. In addition, the pathology in PSP is different to that of PD: In PD there are Lewy Bodies associated with alpha synuclein. In PSP there may be neurofibrillary tangles, as in Alzheimer's disease, as well as tau protein.

Prior structural MRI studies of PSP have examined atrophy of the midbrain [11]–[15]. Using SLD-MRI we here validate the technique with respect to PSP as a disease control.

## Methods

### Ethics Statement

This study was approved by the institutional review committee of the University of Santiago De Chile, and complied with all the internal regulations of the country for its type. It is consistent with good clinical practice and conducted according to the principles that have their origin in the Declaration of Helsinki. All subjects participating in the study provided their written consent, as required by the ethics committee of the University of Santiago De Chile.

### Participants

PSP is a very rare condition. Recruitment of patients was made adhering to internationally recognized diagnostic criteria. The 7 enrolled PSP patients had a gradually progressive form of Parkinsonism, Hoehn and Yahr Stages II–IV and no first degree relatives with PSP or PD. All were over the age of 40. All had vertical gaze palsy, truncal rigidity, and prominent postural instability with falling. All had symmetric examinations and either a poor response, or no response, to levodopa. All were selected from the clinic of a movement disorder specialist (PC). The 7 control subjects were found clinically normal, had no symptoms and no first degree relatives with PD or PSP. The mean age of the PSP patients was 65 years (range 55–72), and control subjects had a mean age of 62 years (range 50–75). The mean duration of the disease was 7 years (range 4–9). An informed consent was obtained from all subjects.

### Magnetic Resonance Imaging

Two heavily T_1_ weighted inversion recovery sequences were used to obtain four slices through the midbrain with white matter suppressed (WMS), and four corresponding slices with gray matter suppressed images (GMS). The WMS image was found to be useful as a template of the SN allowing its isolation from other structures in the mesencephalon (substantia nigra pars reticulata SN_R_, red nucleus and corticospinal tracts). Ratio images (R  =  WMS/GMS) were then computed and the template applied to extract the SN. The middle two slices through the cerebral peduncle were used to generate a quantitative assessment of neurodegeneration.

As described in our previous work [2]–[7], the “lattice” that is responsible for spin-lattice relaxation is taken to be the macromolecular intraneuronal milieu. Since there are gross changes in this milieu with neurodegeneration, the spin-lattice relaxation time T_1_ is expected to be a sensitive measure of such degeneration. The ratio image is designed to be extremely sensitive to small changes in the spin lattice relaxation time T_1_. The WMS image was obtained with a sequence that maximally suppresses signal from the corticospinal tracts, with imaging parameters (TR = 1450 ms, TI = 290 ms, TE = 20 ms). The GMS was designed to maximally suppress the SN, with imaging parameters (TR = 2000 ms, TI = 490 ms and TE = 20 ms) with NEX = 3. The field of view was FOV = 230 mm and the images were 256×256×16 bit matrices.

Four 3 mm thick axial slices perpendicular to the brainstem through the mesencephalon were obtained starting at the pontomesencephalic junction. A gap of 0.2 mm was used. The central two slices were used for image analysis.

### The radiological index RI

The left and right parts of the SN_C_ were divided, by a blinded rater, into lateral and medial sections, as previously applied to IPD patients [2]–[4]. The four mean pixel values (lateral and medial mean values of two sections in two slices) defined the lateral to medial ratio values which were then averaged together over the two slices to yield R_AV_. The radiological index was defined as 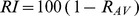.

Though ROI analysis outlining the WMS template of the SN is operator dependent, it was found to be a unique measure to quantitate neurodegeneration in PD patients and was therefore applied to PSP patients.

### The spin-lattice distribution index SI

Recently, a development of this technique was introduced to quantitate neurodegeneration in PD patients in an automatic way. This introduces a spin-lattice distribution index (SI) based on the probability distribution of a histogram of gray level values (frequency of pixel values) within the SN [9]. The procedure was repeated for upper and lower slices of the segmented SN. To give equal weight to both slices, we normalized the frequency distribution of the lower slice to the same area as the upper slice. The two histograms were then added, and finally the resulting area under the frequency distribution curve normalized to 1 for each subject, yielding a grey scale distribution for each subject.

The WMS image is the template for an intramodality data fusion (MRI-MRI) with the ratio image of the SN_C_. The fusion process integrates the WMS substantia nigra and extracts this structure from the ratio image R. The histogram of this final image is then integrated from 50 to 150 to obtain the spin lattice distribution index, SI.

### Image analysis

After extraction of the SN from the cerebral peduncle in the WMS image, the template was applied to the GMS image and the ratio image WMS/GMS of the SN computed. The WMS image of the SN was then used as a background image while the ratio image was used as a foreground image in the fusion process as described in our previous work [5]–[6]. A rainbow color palette was applied to the fused images. A 70% transparency factor was applied to the foreground image as discussed previously [6].

## Results

**Figure 1:**
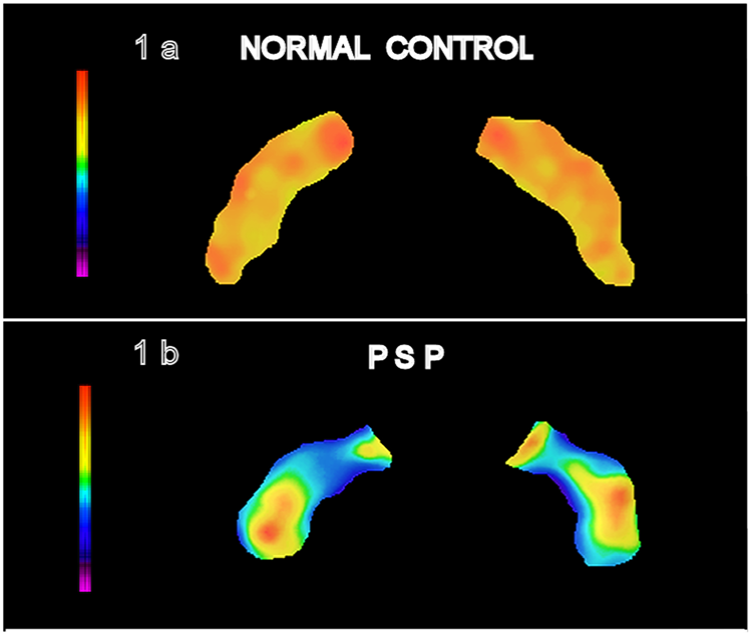
Segmented Substantia Nigra. A representative axial slice through the substantia nigra (SN) is shown for a control subject (1a) and a PSP patient (1b). Note that the medial portions of the nucleus in 1b are more affected (blue signal) than the lateral portions.

Figure 1 shows a representative slice through the substantia nigra for a normal control subject and a PSP patient. In PSP the largest signal change is seen medially, corresponding to the known pattern of pathologic change in PSP, *opposite* to that of PD. This topography reflects the known topography of micropathological changes in the two conditions [10].

### Statistics

Patient and control groups are distinct and without overlap. A nonparametric Kolmogorov-Smirnov test was performed to assess the RI and SI distributions for control subjects and PSP patients. The null hypothesis, that control and PSP groups are indistinguishable is rejected for the RI and SI groups at a significance level p = 0.001. Normal distribution is rejected for the control (C) radiological index data set RI(C), p = 3.47×10^−7^ and for the RI(PSP) data set, p = 2.8×10^−7^. A normal distribution is also rejected for the SI(C), p = 0.038 and for the SI(PSP), p = 0.017.

### Radiological index RI

**Figure 2:**
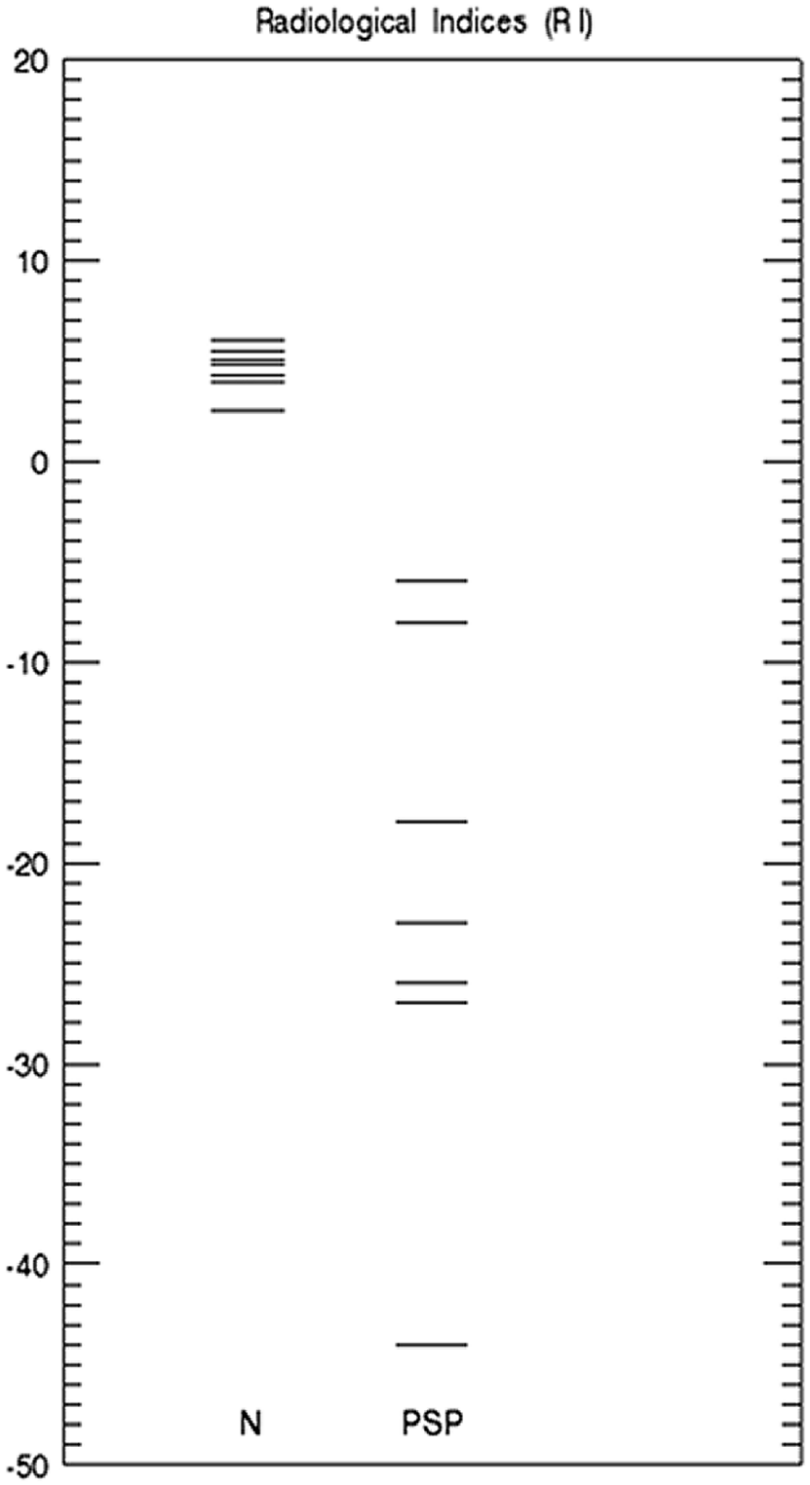
Radiological index. RI is plotted for 7 control subjects (left column labeled N) and 7 PSP patients (labeled PSP). The RI values in controls (N) show small positive values ranging from +2 to +6, while the RI values in PSP patients are within the [−6,−44] interval.

All RIs in PSP patients were therefore negative, as shown in Figure 2. The RI values in normal subjects show a very slightly increased signal in lateral compared to medial sections of the SN_C_, as previously observed [3], [4].

### Spin-lattice distribution index SI

**Figure 3:**
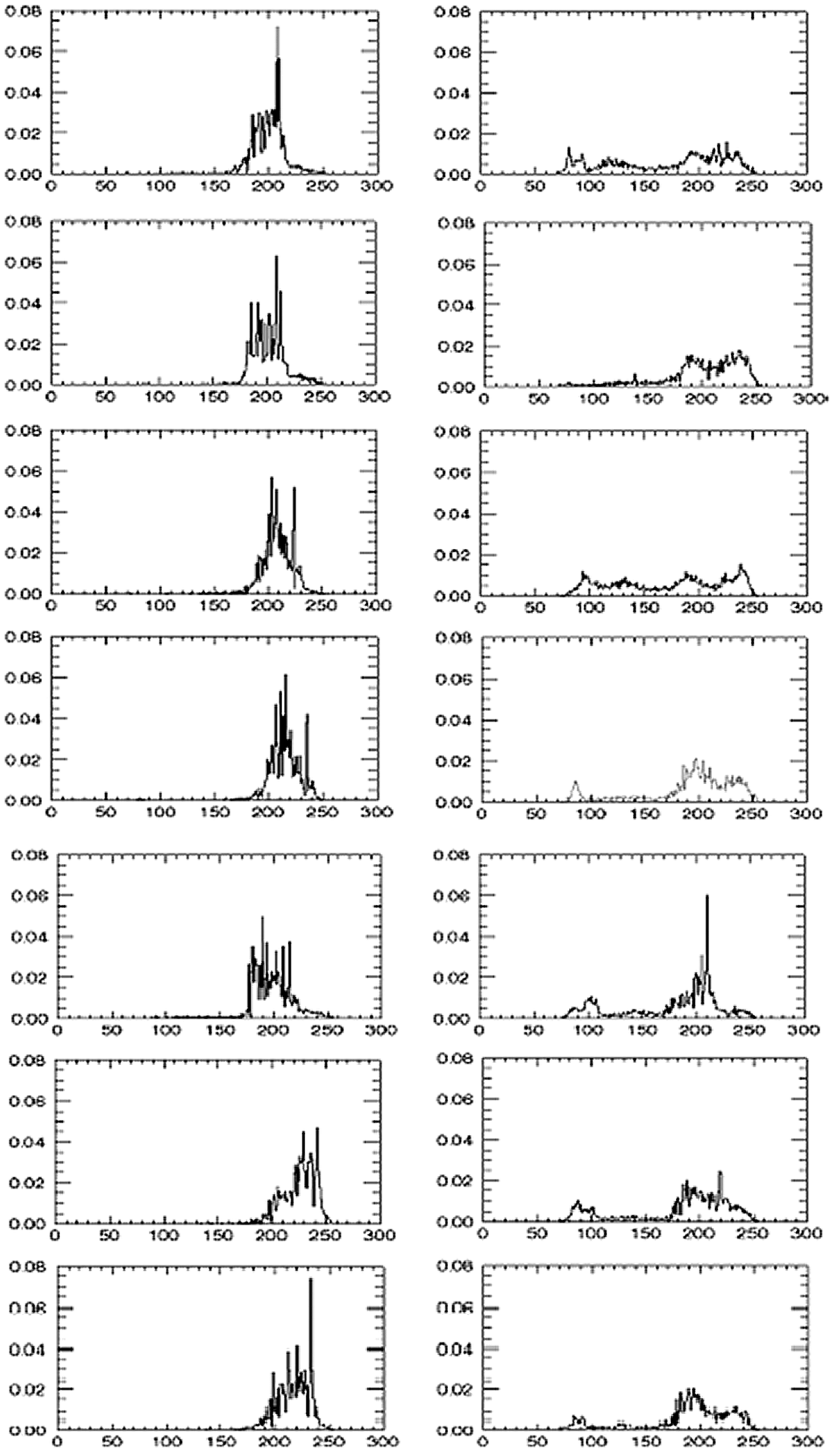
SLD histograms. (gray level distribution normalized to 1) are displayed for the 7 control individuals and 7 PSP patients. The integral from 50 to 150 is then computed to yield the SI. The left column shows the 7 controls and the right column the 7 PSP patients.

The SLD histograms are displayed in Figure 3 for each subject.

**Figure 4:**
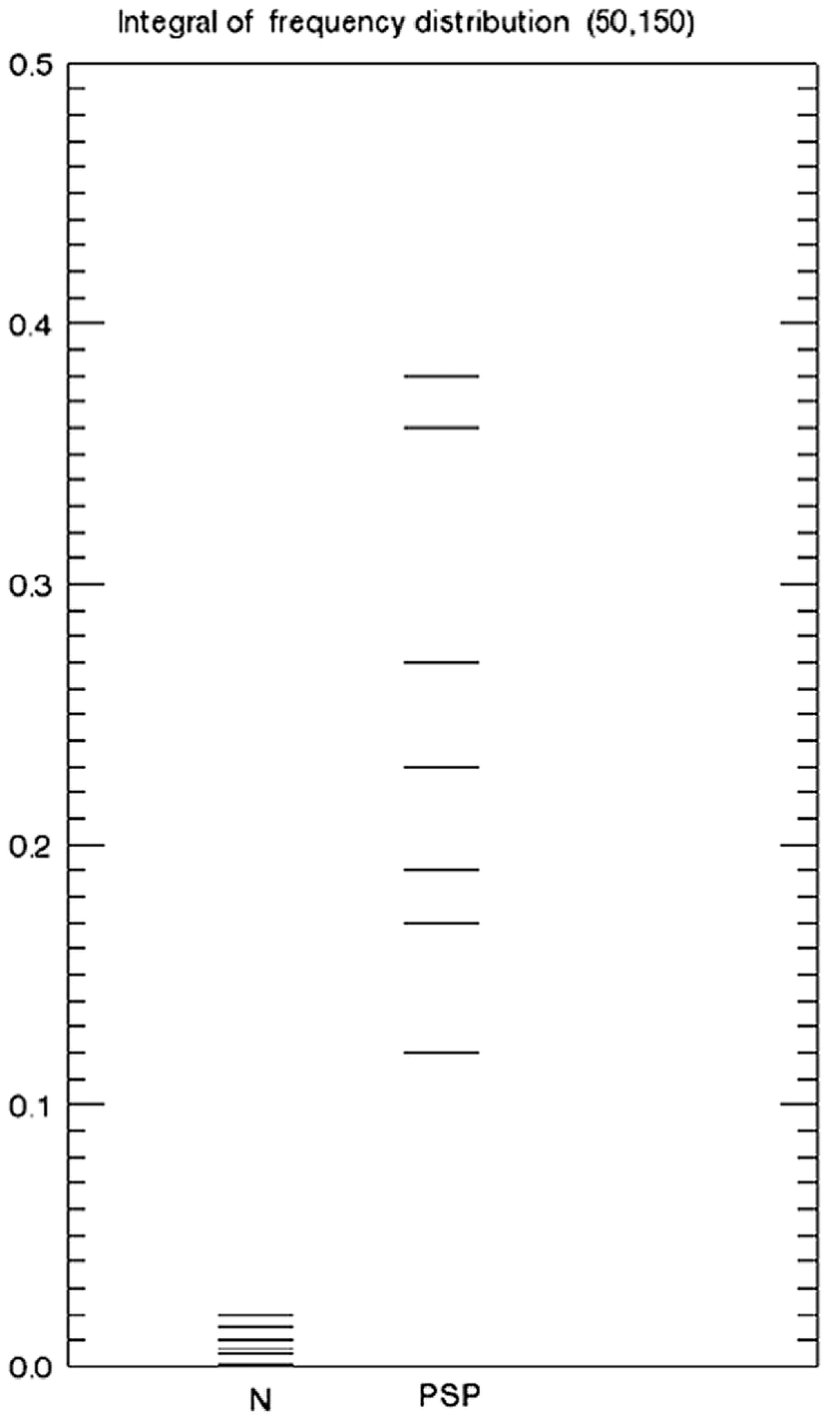
SLD index. SI (integrals from 50 to 150 in Figure 3) is displayed for control subjects (left column labeled N) and PSP patients (right column labeled PSP).

The index SI is displayed in Figure 4 for the controls subjects and PSP patients. SI is the integral of a normalized gray level distribution from 50 to 150 in the standard range [0,255].

Note that SI is positive for all PSP patients, as it is for all patients with Parkinson's Disease [7]. The specificity for PSP therefore arises from the topography of signal change, which can be seen by inspection (Figure 1).

## Discussion

In a group of seven PSP patients and seven control subjects, using precisely the same MRI methodology as had previously been developed for detecting PD [2]–[7], we found a wide separation between patients and control subjects, without overlap, just as we had observed in PD. Moreover, this wide separation - using both RI and SI measures - suggests the possibility of detecting presymptomatic disease.

Of particular note is that negative RI values were seen in all PSP patients, which is the opposite sign to that found in PD [3]–[4]. This radiological finding mirrors the opposite topographies of pathological change in the two conditions [10] and supports the possibility that SLD-MRI may be a biomarker for Parkinsonism at conventional field strengths. Full validation of this technique, however, particularly with respect to sensitivity and specificity in these two forms of Parkinsonism, will be possible only with larger cohorts.

Finally, it is important to remember that we are imaging the substantia nigra, a very small structure in the center of the brain. Anyone attempting to reproduce these images needs to follow the protocol exactly. In particular, the exact same pulse sequences should be used at 1.5 Tesla and, even more importantly, the head must be immobilized. How this is done is not important, although we have found the chinstrap to be a simple, comfortable, and reliable device. If the head is not immobilized, then respiratory movement throughout acquisition leads to volume averaging within the substantia nigra, along with considerable loss of sensitivity.

Anonymized data sets are available from Ulrich Raff PhD, by emailing him at ulrich.raff@usach.cl
